# Effect of melilotus extract on lung injury by upregulating the expression of cannabinoid CB2 receptors in septic rats

**DOI:** 10.1186/1472-6882-14-94

**Published:** 2014-03-11

**Authors:** Ming-wei Liu, Mei-xian Su, Yun-hui Wang, Wei Wei, Lan-fang Qin, Xu Liu, Mao-li Tian, Chuan-yun Qian

**Affiliations:** 1 Department of Emergency, The First Hospital Affiliated To Kunming Medical University, 295 Xichang Road, Wu Hua District, Kunming 650032, China; 2 Intensive Care Unit, The Second Hospital Affiliated To Kunming Medical University, 1 Mayuan, Wu Hua District, Kunming 650106, China; 3 Department of Infectious Diseases, Yan’an Hospital Affiliated to Kunming Medical University, 245 Renmin Eastern Road, Pan Long District, Kunming 650051, China

**Keywords:** Sepsis, Melilotus extract, Cannabinoid receptors, Cecal ligation and puncture, Rats

## Abstract

**Background:**

*M. Suaveolens Ledeb* has long been used in China to treat inflammatory infectious diseases. Melilotus is extracted from *Melilotus Suaveolens Ledeb* and its therapeutic potential is associated with its anti-inflammatory activity. However, the precise mechanisms underlying its effects are unknown. This study was conducted to evaluate the protective effects of melilotus extract in a rat cecal ligation and puncture (CLP)-induced animal model of acute lung injury (ALI).

**Methods:**

A sepsis model was induced by CLP-like lung inflammation. Two hours prior to CLP administration, the treatment group was administered melilotus extract via oral injection. RT-PCR and Western blotting were used to test the expression of cannabinoid receptor (CB)2, NF-κβ and IκB from single peripheral blood mononuclear cells and lung tissues respectively. Enzyme linked immune sorbent assay was used to detect serum levels of tumor necrosis factor (TNF)-α, interleukin (IL)-6, IL-10, and IL-12. The numbers of neutrophils, lymphocytes, macrophages and total cells in the bronchoalveolar lavage (BAL) fluid were counted. For histologic analysis, hematoxylin and eosin (H&E) stains were evaluated.

**Results:**

After inducing ALI by CLP for 24 hours, melilotus extract up-regulated peripheral blood mononuclear cell CB2 expression, blocked the activity of NF-κβ65, and the number of neutrophils, lymphocytes and total cells were significantly lower in the melilotus extract group than the control group. In addition, TNF-α and IL-6 levels were significantly decreased in the melilotus extract group. Histological results demonstrated the attenuation effect of melilotus extract on CLP-induced lung inflammation. CB2 was negatively correlated to NF-κβ mRNA and proteins, respectively (r = -0.377, P < 0.05; r = -0.441, P < 0.05).

**Conclusion:**

The results of this study indicated melilotus extract significantly reduced CLP-induced lung inflammation by up-regulating CB2 expression. The remarkable protective effects of melilotus extract suggest its therapeutic potential in CLP induced-acute lung injury treatment.

## Background

Sepsis, a systemic inflammatory response syndrome (SIRS) induced by infection, is accompanied by the presence of bacteria or a highly suspicious focus of infection [1]. SIRS refers to the systematic inflammatory response leading to a massive, uncontrollable release of inflammatory factors [2]. High levels of inflammatory mediators can lead to the increase of blood capillary permeability and pulmonary edema, resulting in acute respiratory distress syndrome, multiple organ failure, high mortality and other clinical disorders requiring hospitalization. Marijuana (*cannabis sativa*), contains ∆9-tetrahydrocannabinol (THC) as its main active ingredient, and was demonstrated to inhibit cell growth, have anti-inflammatory effects, and restrain tumor growth [3]. There are two subtypes of cannabinoid receptors, CB1 and CB2. CB1 receptors, mainly located in the brain, spinal cord and peripheral nervous system, are related to the regulation of memory, cognition, and motion control; while CB2 receptors are distributed in the peripheral immune system and are closely associated with immune regulation and inhibition of inflammation. Studies indicated that activation of cannabinoid CB2 receptors could regulate the immune system and inhibit inflammation [4]. *M. suaveolens Ledeb* is an annual or biennial melilotus herbage under the *Leguminosae* family, containing a variety of active ingredients, such as coumarin, flavonoids, phenolic acids, steroids, triterpene, and carbohydrates [5]. Studies have shown that M*. suaveolens Ledeb* is the major efficacious factor in *Leguminosae* and is pharmacologically effective in inhibiting inflammation, alleviating pains, detumescence, increasing blood vessel permeability, antibacterial activity, and antiviral action [6]. However, the molecular mechanism of the anti-inflammatory effect of *M. suaveolens Ledeb* is poorly understood. This study developed a cecal ligation and puncture (CLP) rat model of sepsis to observe whether *M. suaveolens Ledeb* could promote the expression of CB2 receptors in rats with sepsis and reduce inflammation, and to determine the impact of *M. suaveolens Ledeb* on sepsis-induced lung injury and its mechanism of action.

## Methods

### Plant material

*M. suaveolens Ledeb* was identified by Prof. Chen Ke-Li (School of Pharmacy, Hubei College of Traditional Chinese Medicine, Wuhan, China) according to Drug Standard of Ministry of Health of the People’s Republic of China (Tibetan Medicine) [7]. Plant materials were stored in the Plant Specimen Department, School of Pharmacy, Hubei College of Traditional Chinese Medicine.

### Preparation of Melilotus extract from *Melilotus suaveolens Ledeb*

Air-dried *M. suaveolens Ledeb* that grew above ground (50 g) was powdered and extracted with 70% ethanol at 85°C (3 × 500 ml, 1.5 hours each). Then the extracted liquid was filtered, combined and concentrated *in vacuo*. Subsequently, the liquid was diluted by deionized water to a concentration of 1 g herb weight per 1 ml water. Further, 5 ml of the liquid was added into a 100 ml separator funnel by a 5 ml Mohr measuring pipette for extraction with 10 ml petroleum ether (60°C–90°C), 10 ml ethyl acetate (EtOAc) and 10 ml n-butanol (BuOH) 6 times for each organic solution, thereby affording a total dry weight of the ethanol extract (EE) of 7.355 g, petroleum ether fraction (PF) of 0.425 g, EtOAc fraction (EF) of 1.425 g, BuOH fraction (BF) of 2.425 g and aqueous fraction (AF) of 3.075 g. Each solution fraction was diluted by 1640 medium to an equal concentration of 5 μg/ml (herb weight per solution volume), which was used to induce CLP-stimulated sepsis.

### High-performance liquid chromatography (HPLC) fingerprint to analyze the herb extract

Since coumarin, rutin and hyperoside were reported as components of the melilotus plant that were anti-inflammatory [8], they were used as standard substances to detect the effects of different solution fractions of ethanol extract from *M. suaveolens Ledeb*. HPLC fingerprint was used to analyze the ingredients of the herb extract [9]. Balance (AB204-N, MAX 210 g, d = 0.1 mg, produced by Merrler Toledo Group) and UV-Detector (8450/HP, Agilent Science of Life and Chemistry Company) were used. The HPLC system consisted of a pump (model DIONEX P680 HPLC Pump, ASI-100 to form a high pressure gradient) with Automated Sample Injector facility, Chromeleon management system and UV–VIS (UVD 170U) model detector. The column was Kromasil C-18 (250 × 4.6 mm, 10 nm-5 mm, Hanbon Science & Technology Co., Ltd). Chromatography conditions included: a gradient elution of acetonitrile and 0.05% H_3_PO_4_ of 1.0 ml min^-1^ flow rate; a 5 ml capacity per injection was used with the UV detector at four wavelengths: 220 nm for coumarin, 254 ± 2 nm, 275 nm coumarin and rutin and 363 nm for hyperoside; the reference concentrations for coumarin, rutin and hyperoside were 0.007648 mg ml^-1^, 0.2548 mg ml^-1^ and 0.2528 mg m^-1^, respectively. The column was placed in a column oven set at 25°C. Then the petroleum ether subfraction was filtered through a 0.45 mm filter membrane (Hanbon Science & Technology Co, Ltd) and stored in a refrigerator before use. The multi-step gradient elution was carried out with acetonitrile and 0.05% H_3_PO_4_ solution. The procedure was as follows: 0–8 min (5% acetonitrile, 95% H_3_PO_4_ solution), 8–25 min (5–30% acetonitrile, 95–70% H_3_PO_4_ solution), 25–35 min (30% acetonitrile, 70% H_3_PO_4_ solution), 35–60 min (30–70% acetonitrile, 70–30% H_3_PO_4_ solution), 60–70 min (30%-5% acetonitrile, 70–95% H_3_PO_4_ solution) and 70–80 min (5% acetonitrile, 95% H_3_PO_4_4 solution).

### Animals

Male Sprague Dawley rats were purchased from Kunming Medical University Laboratory Animal Center (Kunming, China). All rats were housed in the Kunming Medical University animal care facility and were maintained under pathogen-free conditions. Rats were 8–9 weeks of age at the initiation of the experiments and were maintained on standard laboratory chow and water *ad libitum*. All experiments were approved and conducted in accordance with the guidelines of the Animal Care Committee of Kunming Medical University. The experimental procedures were approved by the Ethics Committee of the Institute of Yunan University of Traditional Chinese Medicine (TCM).

### Reagents

Trizol kit was purchased from Gibco, USA; reverse transcription (RT) reaction kit was obtained from Takara Biotechnology Co. Ltd, (Dalian, China); PCR Amplification Reagent Kit and the DNA ladder Marker was acquired from Sangon Biological Engineering Co. Ltd. (Shanghai, China); β-actin was from Santa Cruz Biotechnology, Inc. (USA); TNF-α, IL-6, IL-10 and IL-12 enzyme linked immune sorbent assay (ELISA) kits were from Science and Technology Development Center of the People’s Liberation Army General Hospital, Beijing, China.

### Animal model and group

According to a random number table, 80 rats were randomly divided into 4 groups: normal control group, sham operation group (sham group), sepsis model group (model group) and melilotus treatment group (treatment group), with 20 rats in each group. The sepsis model was induced by CLP. Briefly, animals were deprived of food, but water was permitted for 6 h prior to surgery. Under light ether anesthesia, a laparotomy was performed through a midline abdominal incision. The cecum was punctured twice at different sites with an 18-gauge needle and gently compressed until faces were extruded. The bowel was then returned to the abdomen and the abdominal incision was closed in 2 layers. Animals in the model and treatment groups were treated with 5 ml/100 g body weight of normal saline subcutaneously at the completion of surgery to provide replacement for the extracellular fluid sequestered during peritonitis. Animals in the sham group received sham operation where the cecum was not ligated or punctured. Two hours before surgery, animals in the treatment group received melilotus extract (25 mg/kg) once every eight hours, and the normal control, sham and control groups were given the same volume of saline.

### Sample preparations

After animals in each group were anesthetized with ether at 24 h, the right internal carotid artery was isolated. Blood was extracted (5 ml), centrifuged to collect the supernatant, dispensed into two sterile tubes, sealed with sealing glue, and placed in a freezer at -20°C until used. Extracted peripheral venous blood (2 ml) was placed in EDTA anticoagulant tubes, and peripheral blood mononuclear cells (PBMC) were isolated by Ficoll density gradient centrifugation to detect CB2 expression. All the animals were euthanized 24 h after surgery and samples were collected for further tests.

### RT-PCR analysis

Lung tissue and mononuclear cells were homogenized in TRIzol reagent (Invitrogen) using Mixer 301. Total RNA was extracted according to the manufacturer’s protocol. RNA samples were electrophoresed in agarose gels and visualized with ethidium bromide for quality control. Three micrograms of RNA were reverse transcribed with reverse transcriptase for 1 h at 37°C to synthesize cDNA. Quantitative changes in mRNA expression were assessed by real-time quantitative Real-Time PCR. The PCR master mix contained 0.5 U of Taq polymerase, 1 μL of each primer and 2 μL of each cDNA sample in a final volume of 25 μL. All amplifications were repeated three times. Oligonucleotide primer sequences are shown in Table 1. β2-actin was used as an endogenous control, and each sample was normalized on the basis of its β2-actin content. Relative quantification of mRNA expression levels of target genes was calculated using the 2-∆∆Ct method.

**Table 1:** Primer sequences for genes to RT-PCR validation of microarray analysis

CB2 mRNA	F-5′-GTTCATCGCCTTCCT- 3′	415 bp
	R-5′-CTCGGGGCTTCTTCTTTTG-3′	
NF-κB mRNA	F-5′ -GCACGGATGACAGAGGCGTGTATAAGG-3′	420 bp
	R-5′-GGCGGATGATCTCCTTCTCTCTGTCTG-3′	
IκB mRNA	F-5′-TGCTGAGGCACTTCTGAG-3′	421 bp
	R-5′- CTGTATCCGGGTGCTTGG -3′	
β-actin	F-5′-GATTACTGCTCTGGCTCCTGC-3′	190 bp
	R-5′-GACTCATCGTACTCCTGCTTGC-3′	

### Western blot analysis

Lung tissue and mononuclear cells were homogenized in modified RIPA buffer. Equal amounts of protein (15 μg) were loaded into a 12.5% SDS-polyacrylamide mini-gel, followed by electrophoresis. Protein samples were mixed with sample buffer, boiled for 10 min, separated by SDS-PAGE under denaturing conditions, and electroblotted to nitrocellulose membranes. The blots were incubated overnight in Tris buffered saline (TBS) containing 5% milk to block nonspecific binding of the antibody. Proteins of interest were revealed with specific antibodies as indicated (1:1000 dilution) for 1 h at room temperature followed by incubation with a 1:5000 dilution of horseradish peroxidase-conjugated polyclonal anti-rabbit antibody for 1 h at room temperature. Signals were visualized by chemiluminescence. Equal protein loading of the samples was confirmed by β-actin. All western blots were quantified using densitometry.

### Myeloperoxidase (MPO) activity determination

MPO activities were determined using an MPO kit produced by Jiancheng Bioengineering Institute (Nanjing, China) according to the manufacturer’s instructions. In brief, frozen samples of lung were thawed and homogenized in ice-cold buffer provided in the kit. The homogenates were centrifuged at 5000 × *g* for 10 min. Pellets were suspended in 0.5% hexadecyl trimethyl ammonium bromide in 50 mM PBS (pH 6.0) and incubated at 60°C for 2 h. After another centrifugation, supernatants were collected. Their protein concentrations were measured using a protein assay kit (A045, Jiancheng Bioengineering Institute). In a 96-well plate, 15 μg protein was incubated with 100 μl 3,3R,5,5R-tetramethylbenzidine for 3 min. After 100 μl sulfuric acid (1 N) was added, absorbance was read by a spectrophotometer at a wavelength of 450 nm. Original MPO values were normalized with protein contents.

### Determination of levels of inflammatory cells in bronchoalveolar lavage fluid (BAL)

A tracheotomy was performed in 25% urethane-anesthetized animals, by inserting a 5 mm plastic tube, and repeating lavage five times with saline and a gauze filter. Samples were immediately stored at 4°C until further processing within 2 hours of the BAL procedure. BAL fluid was strained through a monolayer of surgical gauze to remove mucus. An aliquot was reserved for total cell counts. The total cell number was counted using a Nageofte’s chamber, and results were expressed as cells × 10^3^/ml. The remaining fluid was immediately centrifuged at 800 × *g* for 10 mm at 4°C and the cell pellet was washed twice with phosphate-buffered saline solution (without Ca^++^ and Mg^++^). Cytocentrifugates (Labofuge, AE Meraeus, FRG) were stained using the May-Grunwald-Giemsa method. The differential cell count of macrophages, lymphocytes, neutrophils and eosinophils was made under light microscopy at × 1000 magnification, by counting approximately 300 cells in random fields. Because only a few mast cells were present and difficult to detect using the May-Grunwald-Giemsa stain, they were counted in cell suspension after staining with Alcian blue-safranine. Mast cells were quantified by counting 1,000 cells. Results were expressed as the number of cells × 10^6^/L of recovered fluid.

### Cytokine measurement in serum

TNF-α, IL-12, IL-6, IL-4, and IL-10 serum levels of rats was measured by ELISA according to the manufacturer’s instructions (R&D Systems).

### Histopathologic examination of lung tissue

Lungs from all four groups were excised at 24 h following the challenge dose of endotoxin. The lungs were fixed with 100% ethanol intratracheally under 20 cm of H_2_O pressure. After fixation, lungs were embedded in paraffin, cut into 5-μm sections, and stained with hematoxylin and eosin (HE). Lung sections were evaluated and scored independently by 2 members of the study group trained in histological assessment and use of the scoring system. For each mouse, 3 different lobes were examined for the following features: interstitial edema, hemorrhage, and neutrophil infiltration. Each feature was scored as follows: 0 (no injury), 1 (minimal injury), 2 (moderate injury), or 3 (severe injury). This was totaled to provide an individual lobe score, and the mean of the 3 lobes was used to generate a score for each mouse, giving a minimum score of 0 and a maximum of 9.

### Survival Curves

Another 45 rats were divided into sham operation group, CLP group, and CLP plus melilotus extract group (n = 15 per group) to observe survival. The treatments were the same as for the previous experiments. Observation was begun at the time of melilotus extract treatment, while the endpoint was set at 120 hours after melilotus extract treatment.

### Statistical analysis

SPSS 11.0 software was used for statistical analysis of the results. Data were expressed as the mean ± SD. Statistical differences between the two groups were evaluated by analysis with *t*-test; analysis of linear correlation was used to evaluate the correlation between two variances; q-test of analysis of variance (ANOVA) was used to analyze multiple comparison. Survival data were analyzed utilizing log-rank or χ2. Values of P < 0.05 were accepted as indicating significance.

## Results

### HPLC fingerprint of ethanol extract from M. suaveolens Ledeb

**Figure 1:**
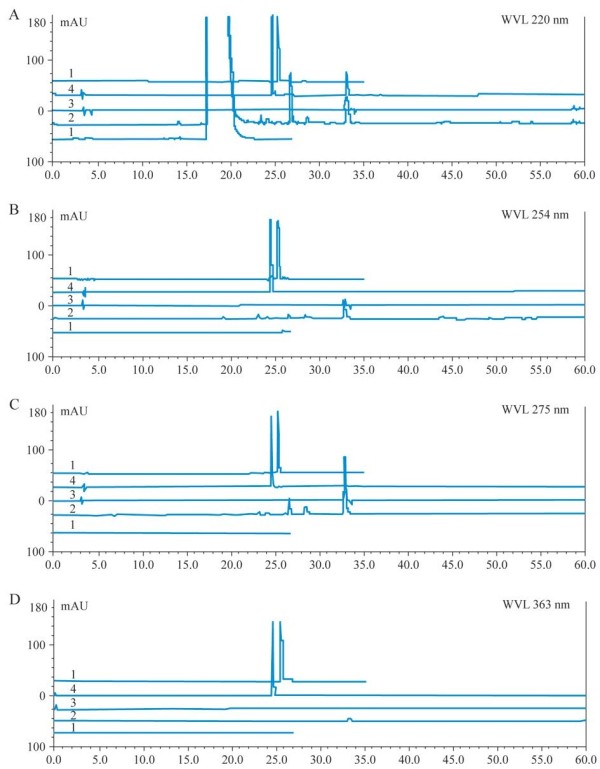
**HPLC fingerprint of the EtOAc fraction of M. suaveolens Ledeb at 220 nm (A), 254 nm (B), 275 nm (C) and 363 nm (D).** (Note: line 1 represents blank control of EtOAc solvent, line 2 represents standard substances of coumarin, line 3 represents rutin and line 4 represents rutin and hyperoside).

HPLC fingerprint analysis (Figure 1) shows *M. suaveolens Ledeb* EtOAc fraction had coumarin (retention time was 33.225 min), but no hyperoside or rutin (retention times were 24.535 min and 25.407 min, respectively). Thus, coumarin might be an anti-inflammatory compound present in the *M. suaveolens Ledeb* EtOAc fraction, and changes in coumarin content in different intervention liquids could explain their different effects (Tables 2 and 3). Coumarin was chosen as a reference peak in HPLC fingerprint analysis and the relative retention time (RT) and relative area (RA,%) of the EtOAc fraction of ethanol extract were calculated (Table 4). In addition, this data suggested the *M. suaveolens Ledeb* EtOAc fraction mainly contained lower polar compounds including coumarin.

**Table 2:** Contents of coumarin in different analytical liquids (n = 3)

**Sample**	**Concentration of the liquid (mg/ml)**	**Coumarin in the analysis liquid (μg/ml)**
Ethanol extract (EE)	12	11.4990
Petroleum ether fraction (PF)	150	42.0393
EtOAc fraction (EF)	75	48.6013
BuOH fraction (BF)	150	3.4989
Aqueous fraction (AF)	150	0
Coumarin		0.0075

**Table 3:** Contents of coumarin in different intervention liquids (n = 3)

**Sample**	**Concentration of intervention liquid (ng/ml)**
Ethanol extract (EE)	4.891
Petroleum ether fraction (PF)	1.4110
EtOAc fraction (EF)	3.1902
BuOH fraction (BF)	0.1096
Aqueous fraction (AF)	0
Coumarin	

**Table 4:** RT and RA (%) of various peaks in analytical liquid HPLC fingerprint

RT	0.429	0.695	0.729	0.810	0.870	1.000	1.580	1.803
RA (%)	2.300	2.839	3.197	30.713	3.841	51.104	4.001	1.897

### Effect of melilotus extract on the expression of CB2 in mononuclear cells in peripheral blood, NF-κβ, and IκB in lung tissue by RT-PCR and western blotting

**Figure 2:**
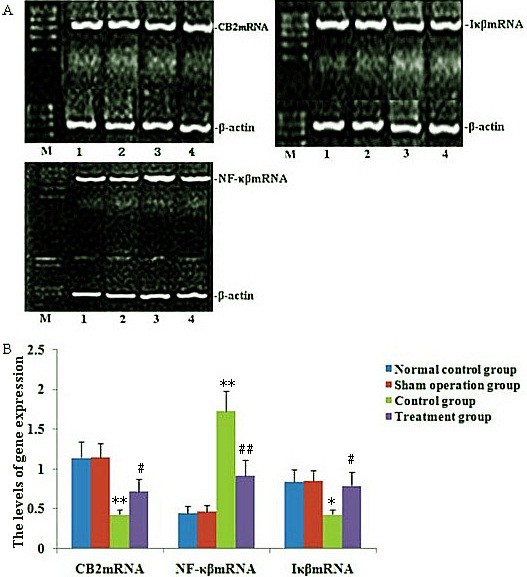
**Effect of melilotus extract on the expression of CB2 mRNA in mononuclear cells from peripheral blood, NF-κβ mRNA and IκB mRNA from lung tissue. A)**: Representative RT-PCR showing the level of CB2 mRNA, NF-κβ mRNA and Iκβ mRNA expression in rats at 24 h after tubed administration of melilotus extract. 1: mark; 2: normal control group; 3: sham operation group; 4: control group; 5: treatment group. **B)**: Statistical summary of the densitometric analysis of CB2 mRNA, NF-κβ mRNA and Iκβ mRNA expression in rats. Each value represents the mean ± SD as determined from three independent experiments. *P < 0.05, **P < 0.01, vs the normal control group and sham operation group; #P < 0.05, ##P < 0.01, vs the control group.

**Figure 3:**
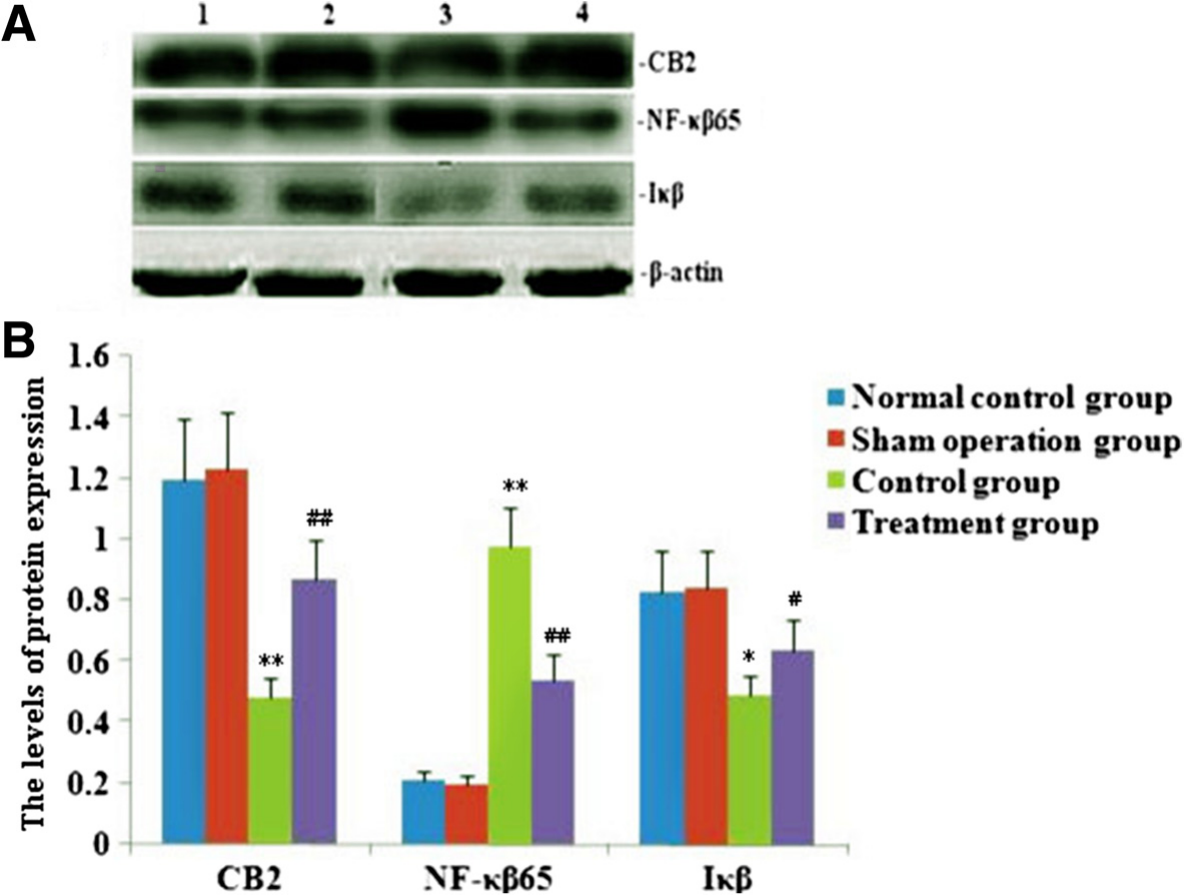
**Effect of melilotus extract on the expression of CB2 in mononuclear cells in peripheral blood, NF-κβ and Iκβ in lung tissue by western blot. A)**: Representative western blots show the level of CB2, NF-κβ and Iκβ expression in rats at 24 h after tubed administration of melilotus extract. 1: normal control group; 2: sham operation group; 3: control group; 4: treatment group. **B)**: Statistical summary of the densitometric analysis of CB2, NF-κβ and Iκβ expression in rats. Data represent the mean ± SD of 3 experiments. *P < 0.05, **P < 0.01, vs the sham operation group; ##P < 0.01, #P < 0.01, vs the control group.

**Figure 4:**
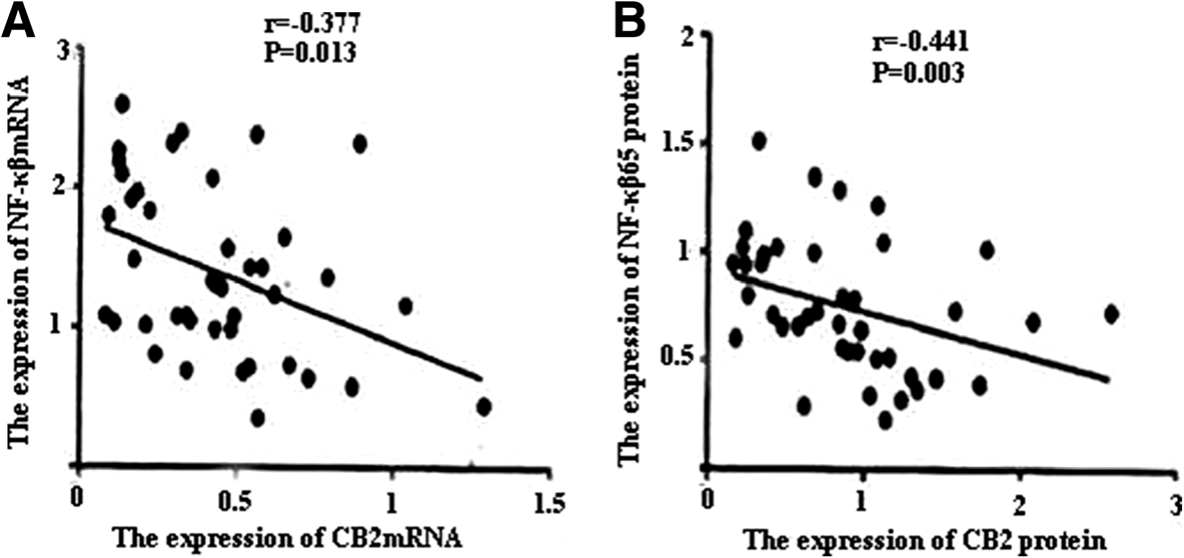
**Change in correlation between CB2 and NF-κβ mRNA and protein expression. A)**: Statistical summary of correlation between CB2 mRNA and NF-κβ mRNA expression. **B)**: Statistical summary of correlation between CB2 and NF-κβ expression. Analysis of linear correlation was used to evaluate the correlation between CB2 and NF-κβ mRNA and protein. A negative correlation exists between CB2 mRNA and NF-κβ mRNA as well as between CB2 and NF-κβ protein (r = -0.377, P < 0.05; r = -0.441, P < 0.05).

PT-PCR and western blotting were used to measure the expressions of CB2, NF-κβ and IκB within single PBMC and lung tissues. RT-PCR and western blot analysis found that the expression of CB2 and IκB was markedly decreased, and NF-κβ expression was significantly up-regulated after CLP. After melilotus extract therapy, the expression of CB2 and Iκβ was markedly enhanced, while NF-κβ expression was significantly lowered. Therefore, melilotus extract could suppress the expression of NF-κβ, and up-regulate CB2 and IκB expression (Figures 2 and 3). A negative correlation existed between CB2 mRNA and NF-κβ mRNA as well as between CB2 and NF-κβ (r = -0.377, P < 0.05; r = -0.441, P < 0.05) (Figure 4).

### Effect of melilotus extract on levels of TNF-α, IL-6, IL-10, IL-4, and IL-12 in serum

**Figure 5:**
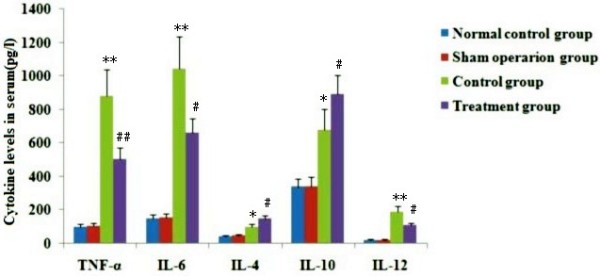
**Effect of melilotus extract on the levels of TNF-α, IL-6, IL-10, IL-4, and IL-12 in plasma.** Plasma TNF-α, IL-4, IL-6, IL-10 and IL-12 levels were measured by ELISA. The data represent the means ± SD (n = 20, pg/L) from triplicate experiments. **P < 0.01, *P < 0.05, vs the sham operation group; #P < 0.05, ##P < 0.01, vs the control group.

Serum TNF-α, IL-6, IL-12, IL-4, and IL-10 levels were increased significantly in CLP-induced rats. After administration with melilotus extract, the levels of proinflammatory cytokines (TNF-α, IL-12 and IL-6) were significantly decreased, while levels of anti-inflammatory cytokines (IL-10 and IL-4) were markedly increased (Figure 5).

### Effect of melilotus extract on MPO activity in lung tissue

**Figure 6:**
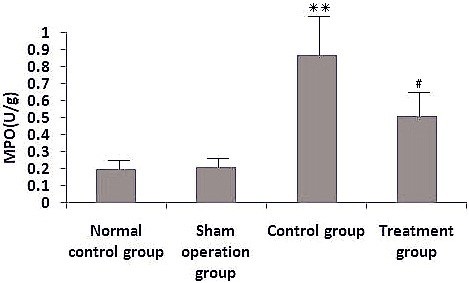
**Effect of melilotus extract on MPO levels in lung tissues.** MPO activities were determined using an MPO kit. Data are expressed as the mean ± SD as determined from three independent experiments. **P<0.01, vs the sham operation group; #P<0.05, vs the control group.

After cecal ligation and puncture operation, MPO activity in lung tissue was obviously increased. After administration with melilotus extract, MPO activity was significantly decreased (Figure 6).

### Effect of melilotus extract on the number of inflammatory cells in BAL fluid

**Figure 7:**
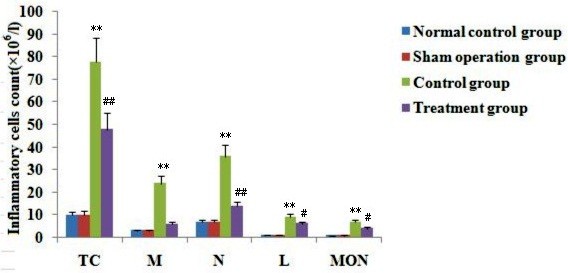
**Effect of melilotus extract on inflammatory cells in BAL fluid.** TC: total inflammatory cells, MAC: macrophages, N: neutrophils, L: lymphocytes, MON: monocytes. Inflammatory cell counts are expressed as the mean ± SD (n = 20, × 10^6^/L) as determined from three independent experiments. **P < 0.01; vs the sham operation group; #P < 0.05, ##P < 0.01, vs the control group.

The total inflammatory cell, macrophage, neutrophil, lymphocyte and monocyte counts in BAL fluid in the CLP group were obviously increased. After melilotus extract treatment, the total inflammatory cell, macrophage, neutrophil, lymphocyte and monocyte counts in BAL fluid were significantly decreased (Figure 7).

### Effect of melilotus extract on histopathologic changes of lung tissue

**Figure 8:**
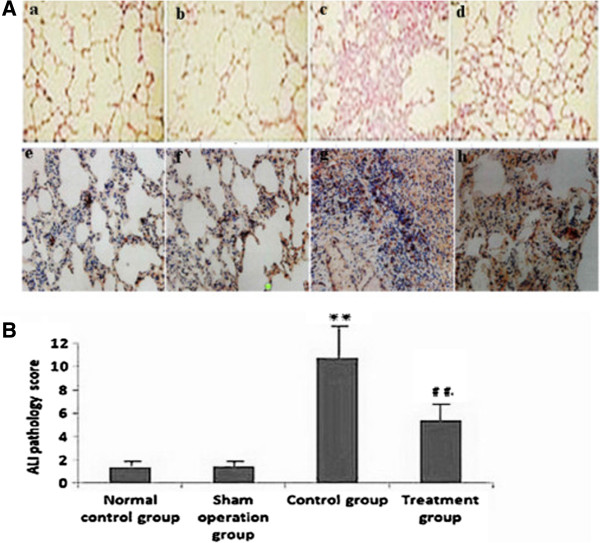
**Histopathological changes of lung tissues in rats (a → d, HE staining, ×100 magnification; e → h HE staining, ×400 magnification. A)**: Histopathological changes in lung tissues from rats a: normal control group, b: sham operation group, c: control group, d: treatment group, e: normal control group, f: sham operation group; g: control group, h: treatment group. **B)**: Statistical summary of ALI pathology score in rats. ALI pathology scores were expressed as the mean ± SD from triplicate experiments. **P < 0.01; vs the sham operation group; ##P < 0.01; vs the control group.

There was no sign of hemorrhage, edema and inflammatory cell infiltration in lung tissue in the normal and sham groups. After CLP, hemorrhage, edema and infiltration of inflammatory cells were observed in lung tissues, and acute lung injury (ALI) pathology scores were significantly enhanced compared with the model group. In contrast, injury to lung tissue was alleviated in the melilotus treatment group and ALI pathology scores were significantly reduced (treatment group) (Figure 8).

### Effect of melilotus extract on survival of sepsis rats

**Figure 9:**
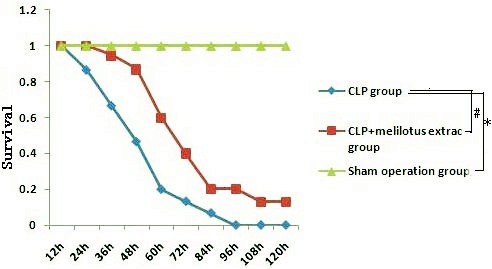
**Survival curve.** CLP group: group challenged with CLP; CLP + melilotus extract groups: group challenged with CLP and treated with melilotus extract. *P < 0.05, vs sham operation group. #P < 0.05; vs CLP group.

The survival of rats was markedly decreased in rats receiving CLP compared to the control group. The decreased survival induced by CLP was significantly attenuated by post-treatment with melilotus extract as compared to the CLP-induced alone group (Figure 9).

## Discussion

Abundant evidence shows that CLP-induced sepsis with acute suppurative peritonitis is a typical sepsis model with G-bacteria as the predominant infection source [6]. The CLP sepsis model is highly stable, repetitive, and applicable, thus it is currently regarded as the “golden standard” for sepsis-related studies. This model, by virtue of cecal ligation and perforation, leads to the pollution of the abdominal cavity by bacteria-carrying intestinal contents, gives rise to generalized peritonitis, and induces a wide range of systemic inflammatory responses [10]. Furthermore, model animals initially demonstrate clinical effects resembling high power cycle and high metabolism, and a low power cycle state at later stages. Thus, it is regarded as the sepsis model with the strongest clinical relevance [11]. Previous studies showed that approximately 18–72 h after cecum ligation perforation, rats exhibit lung injury, low-oxygen, neutrophil cell infiltration, and alveoli and interstitial edema [12]. Therefore, the CLP animal model of rats with sepsis was used in this study.

Marijuana has been used for medicine for thousands of years. Δ9-THC is the major active constituent of marijuana. As proposed by Kunos, [13] multiple cannabinoid receptors have been demonstrated throughout the body, but only CB1 and CB2 are commonly studied. CB1 is mainly expressed in the brain, spinal cord and peripheral nervous system, mainly related to the regulation of memory, cognition, and motion control; while CB2 does not exist in the central nervous system [14], but instead is distributed in the peripheral immune system, including B cells, natural killer cells, monocytes, macrophages, neutrophils, dendritic cells, mast cells and T cells [15]. Studies have shown that CB2 receptors are mainly involved in the *in vivo* immune response and can inhibit inflammation. The major actions are as follows: (1) inhibition of endothelial cell inflammatory responses [16]; (2) inhibition of monocyte migration and inflammatory cytokine release [9]; (3) inhibition of proliferation and migration of vascular smooth muscle; and (4) inhibition of the proliferation of T cell activation [17]. For example, the selective CB2 receptor stimulant AM1241 can act on CB2 receptors on peripheral immune cells, inhibiting the release of inflammatory factors and easing pain [18]. Importantly, CB2 agonists do not induce side effects in the central nervous system. CB2 receptors can regulate B cell and T cell differentiation as well as the balance between the proinflammatory and anti-inflammatory effects of Thl/Th2 helper T cells. The activation of CB2 receptors can also inhibit macrophage proliferation and reduce the release of proinflammatory cytokines TNF-α, nitric oxide, and IL-12p40 [16]. Rajesh et al. [18] found that JWH-133, a specific CB2 receptor activator, inhibited TNF-α-induced expression of intercellular adhesion molecule-1, vascular cell adhesion molecule 1, macrophage chemotactic protein-1, signaling pathway activation of nuclear factor, and the migration of monocytes to arterial endothelial cells. In the current study, increased CB2 expression inhibited NF-κβ expression, reduced the produce of proinflammatory factors (TNF-α and IL-6), decreased MPO production and the accumulation of inflammatory cells, and significantly alleviated lung injury.

It was shown that through involvement in the transcription of a variety of cytokine genes, NF-κβ could exert a complicated impact on the regulating network. Activated NF-κβ can enhance the transcription of many cytokines, such as TNF-α and IL-1, [19], to lengthen the synthesizing time and quantity of inflammation factors [20]. Inhibition of NF-κβ activity can decrease the expression of inflammatory media regulated by NF-κβ, and reduce the infiltration and activation of inflammatory cells, thus protecting lung tissues from damage [21]. IκB also has a critical role in the transcription and expression of inflammatory factors regulated by NF-κβ [22]. The current study indicates that inhibition of NF-κβ expression or promotion of IκB expression can both be conducive to the reduction of proinflammatory factors (TNF-α and IL-6), the generation of anti-inflammatory media (IL-10 and IL-12), the inhibition of accumulation of inflammatory cells, production of MPO within the lung, and alleviation of lung damage.

As a traditional Chinese medicine, *M. suaveolens Ledeb* is an annual or biennial melilotus herbage under the *Leguminosae* family. It tastes bitter and cold, and is functional in heat-clearing and detoxifying, anti-inflammation and detumescence. Thus, it can be applied to splenic disease, twisted intestinal fever, diphtheria, and tonsillitis amongst others [23]. Pharmaceutical study results indicated *M. suaveolens Ledeb* contains several substances such as coumarin, flavonoids, phenolic acids, and saponins with anti-inflammatory and anti-bacterial properties [23]. HPLC fingerprint analysis of the herb extract showed that the only content of petroleum ether extract from *M. suaveolens Ledeb* was coumarin. Pharmacological studies showed that melilotus extract tablets reduced vascular permeability, enhanced capillary strength, inhibited loss of serum proteins, maintained normal colloid osmotic pressure, reduced leakage, and had a diuretic effect by inhibiting renal tubular reabsorption of sodium and chlorine [24], thus alleviating or eliminating inflammation and edema [25]. In addition, they could promote protein decomposition in tissues, activate protein hydrolysis of macrophages, reduce protein concentration, reduce osmotic pressure, and promote lymphatic circumfluence [26], thus reducing tissue edema. Moreover, these factors could effectively inhibit the biosynthesis and release of inflammatory factors such as opioid peptides and β-endorphin to alleviate inflammation, which, therefore, has obvious anti-inflammatory and analgesic effects. Asres K, et al. [27] used a granulation swelling experiment to confirm that both melilotus suaveolens extract and melilotus suaveolens preparations have powerful anti-inflammatory effects. Plesca-Manea et al. [28] demonstrated that extracts from melilotus suaveolens with 0.25% coumarin and hydrocortisone sodium were similarly effective as anti-inflammatory agents. The anti-inflammation effect may be attributed to the reducing effects of melilotus suaveolens extract on the activation of phagocytic cells and generation of citrulline. Ritschel et al. [29] showed that intravenous injection of 12.5–100 mg/kg of coumarin to rats had dose-related anti-inflammatory and detumescence effects. Furthermore, subcutaneous injection of coumarin alleviated the symptoms of formaldehyde-induced edema in rats. *In vitro* studies have shown that different extracts and coumarin from *melilotus suaveolens* can inhibit lipopolysaccharide stimulation of RAW264.7 cells to generate proinflammatory factors, such as IL-6, TNF-α, IL-1β, and NO, and can improve the generation of the anti-inflammatory factor, IL-10 [30]. Thus, it can be seen from this study that melilotus suaveolens can promote the expression of CB2, inhibit the expression of NF-κβ, increase Iκβ expression, reduce the generation of proinflammatory factors (TNF-α, IL-12, and IL-6), increase the production of anti-inflammatory factors (IL-10 and IL-4), inhibit MPO production and the accumulation of inflammatory cells within the lung, and alleviate lung injury. Therefore, melilotus suaveolens can inhibit inflammatory cell invasion and significantly alleviate lung injury, thus having a protective role for lungs.

## Conclusions

In summary, this study clearly demonstrated the anti-inflammatory effect of melilotus extract via increased cannabinoid CB2 receptor activation. Moreover, melilotus extract blocked CLP-induced NF-κβ expression and up-regulated Iκβ expression, inhibited MPO production. It also reduced the accumulation of inflammatory cells within the lung, and alleviated the pathological injury of lung tissues in rats with sepsis, which provides an animal experimental basis for the treatment of sepsis by application of *M. suaveolens Ledeb*.

## Abbreviations

CB2: Cannabinoid CB2 receptors; MPO: Myeloperoxidase; ALI: Acute lung injury; NF-κB: Nuclear factor kappa B; IL-6: Interleukin-6; IL-10: Interleukin-10; IL-12: Interleukin-12; TNF-α: Tumor necrosis factor-α; BALF: Bronchoalveolar lavage fluid; TCM: Traditional Chinese Medicine; PBS: Phosphate-buffered saline; HPLC: High-performance liquid chromatography; RT-PCR: Reverse transcription polymerase chain reaction; PBMC: Peripheral blood mononuclear cells; CLP: Cecal ligation and puncture; SIRS: Systemic inflammatory response syndrome; THC: ∆9-tetrahydrocannabinol.

## Competing interests

All the authors declare no conflict of interest relevant to the subject matter or materials discussed in the article.

## Authors’ contributions

M-WL, Y-HW, XL and M-LT have made contributions to the acquisition and analysis of data. L-FQ, WW and M-XS were involved in the interpretation of data. M-WL, M-XS and C-YQ were involved in designing the study and drafting the manuscript. All authors read and gave final approval for the version submitted for publication.

## Pre-publication history

The pre-publication history for this paper can be accessed here:

http://www.biomedcentral.com/1472-6882/14/94/prepub

## References

[B1] Lee YS Kim SY Kwon CW Song HG Lee YK Kim HJ Two cases of systemic capillary leak syndrome that were treated with pentastarch Korean J Intern Med 2007 22 130 132 10.3904/kjim.2007.22.2.130 17616032 PMC2687610

[B2] Zhao L Tao JY Zhang SL Jin F Pang R Dong JH N-butanol Extract from Melilotus Suaveolens Ledeb Affects Pro- and Anti-Inflammatory Cytokines and Mediators Evid Based Complement Alternat Med 2010 7 97 106 10.1093/ecam/nem165 18955281 PMC2816390

[B3] Galiègue S Mary S Marchand J Dussossoy D Carrière D Wang Z Expression of central and peripheral can nabinoid receptors inhuman immune tissues and leukocyte subpopulations Eur J Biochem 1995 232 54 61 10.1111/j.1432-1033.1995.tb20780.x 7556170

[B4] Kieman JA Histologic and histochemical Theory and practice 2008 4 Bloxham, UK: Scion

[B5] Kruger NJ Walker J The Bradford method for protein quantitation The protein protocois handbook 2002 Hatfield UK: University of Herfordshire 15 21

[B6] Seely KA Holthoff JH Burns ST Wang Z Thakali KM Gokden N Rhee SW Mayeux PR Hemodynamic changes in the kidney in a pediatric rat model of sepsis-induced acute kidney injury Am J Physiol Renal Physiol 2011 301 F209 F217 10.1152/ajprenal.00687.2010 21511700 PMC3129882

[B7] Sichuan Ganzi Institute for Drug Control China Pharmacopoeia Committee Melilotus suaveolens Ledeb Drug Standard of Ministry of Health of the People’s Republic of China (Tibetan Medicine) 1995 1 Beijing: People’s Medical Publishing House 65

[B8] Qi LW Yu QT Li P Li SL Wang YX Sheng LH Yi L Quality evaluation of Radix astragali through a simultaneous determination of six major active isoflavonoids and four main saponins by highperformance liquid chromatography coupled with diode array and evaporative light scattering detectors J Chromatogr A 2006 1134 162 169 10.1016/j.chroma.2006.08.085 16982063

[B9] Ziring D Wei B Velazquez P Schrage M Buckley NE Braun J Formation of B and T cell subsets require the cannabinoid receptor CB2 Immunogenetics 2006 58 714 725 10.1007/s00251-006-0138-x 16924491

[B10] Ritter C Andrades M Frota Júnior ML Bonatto F Pinho RA Polydoro M Klamt F Pinheiro CT Menna-Barreto SS Moreira JC Dal-Pizzol F Oxidative parameters and mortality in sepsis induced by cecal ligation and perforation Intensive Care Med 2003 29 1782 1789 10.1007/s00134-003-1789-9 12783160

[B11] Uji Y Yamamoto H Tsuchihashi H Maeda K Funahashi T Shimomura I Shimizu T Endo Y Tani T Adiponectin deficiency is associated with severe polymicrobial sepsis, high inflammatory cytokine levels, and high mortality Surgery 2009 145 550 557 10.1016/j.surg.2009.01.010 19375615

[B12] Bello GM Frevert CW Martin TR Animal models of acute lung injury Am J Physiol Lung Cell Mol Physiol 2008 295 379 397 10.1152/ajplung.00010.2008 PMC2536793 18621912

[B13] Kunos G László SB Offertáler L Mo F Liu J Karcher J Harvey-White J The quest for a vascular endothelial cannabinoid receptor Chem Phys Lipids 2002 121 45 56 10.1016/S0009-3084(02)00145-7 12505689

[B14] Romero-Sandoval A Nutile-McMenemy N DeLeo JA Spinal microglial and perivascular cell cannabinoid receptor type 2 activation reduces behavioral hypersensitivity without tolerance after peripheral nerve injury Anesthesiology 2008 108 722 734 10.1097/ALN.0b013e318167af74 18362605 PMC2647363

[B15] Romero-Sandoval A Eisenach JC Spinal cannabinoid receptor type 2 activation reduces hypersensitivity and spinal cord glial activation after paw incision Anesthesiology 2007 106 787 794 10.1097/01.anes.0000264765.33673.6c 17413917

[B16] Held-Feindt J Dörner L Sahan G Mehdorn HM Mentlein R Cannabinoid receptors in human astroglial tumors J Neurochem 2006 98 886 893 10.1111/j.1471-4159.2006.03911.x 16893424

[B17] Stebulis JA Johnson DR Rossetti RC Burstein SH Zurier RB Ajulemic acid, asynthetic cannabinoid acid, induces an antiinflammatory profile of eicosanoids in human synovial cells Life Sci 2008 83 666 670 10.1016/j.lfs.2008.09.004 18840450

[B18] Rajesh M Mukhopadhyay P Haskó G Huffman JW Mackie K Pacher P CB2-receptor stimulation attenuates TNF-alpha-induced human endothelial cell activation, transendothe lia lm-igration of monocytes, and monocyte-endothelial adhesion Am J Physiol Heart Circ Physiol 2007 293 H2210 H2218 10.1152/ajpheart.00688.2007 17660390 PMC2229632

[B19] Ramos G Limon-Flores AY Ullrich SE JP-8 induces immune suppression via a reactive oxygen species NF-κβ–dependent mechanism Toxicol Sci 2009 108 100 109 10.1093/toxsci/kfn262 19095747 PMC2721654

[B20] Huang Y Nikolic D Pendland S Doyle B Locklear T Mahady G Effects of cranberry extracts and ursolic acid derivatives on P-fimbriated Escherichia coli, COX-2 activity, pro-inflammatory cytokine release and the NF-κβ transcriptional response in vitro Pharm Biol 2009 47 18 25 10.1080/13880200802397996 20376297 PMC2849675

[B21] Zingarelli B Sheehan M Hake PW Connor MO Denenberg A Cook JA Peroxisome Proliferator Aetivator Receptor-γ Ligands,15-Deoxy-△^12,14^- Prostaglandin J2 and Cigitazone, reduce systemic inflammnationin polymierobial sepsis by modulation of signal transduction pathways J Immunol 2003 171 6827 6837 14662889 10.4049/jimmunol.171.12.6827

[B22] Guo XH Pan YF Xiao CY Wu YQ Cai DZ Gu JR Fractalkine stimulates cell growth and increases its expression via NF-κB pathway in RA-FLS Int J Rheum Dis 2012 15 322 329 10.1111/j.1756-185X.2012.01721.x 22709495

[B23] Gebre MT Asres K Getie M Endale A Neubert R Schmidt PC In vitro availability of kaempferol glycosides from cream formulations of methanol extract of the leaves of Melilotus elegans Eur J Pharm Biopharm 2005 60 31 38 10.1016/j.ejpb.2005.01.001 15848053

[B24] Saxena VK Nigam S Antifungal studies of pterocarponoids from Melilotus indica Asian J Chem 1996 8 337 338

[B25] Foidi M Zoltan OI Learning ability in experimental lymphogenic encephalopathy under the influence of coumarin from Melilotus officinalis Arzneimittelforschung 1970 20 1614 1635 5536958

[B26] Kang SS Lee YS Lee EB Saponins and flavonoid glycosidesfrom yellow sweet clover Arch Pharm Res 1988 11 197 202 10.1007/BF02861309

[B27] Asres K Gibbons S Nachname Vorname Anti-inflammatory activity of extracts and a saponin isolated from melilotu selegans Pharmazie 2005 60 310 312 15881614

[B28] Plesca-Manea L Parvu AE Parvu M Tarmak M Buia R Puia M Effects of Melilotusofficinalis on acute inflammation Phytother Res 2002 16 316 10.1002/ptr.875 12112285

[B29] Ritschel WA Brady ME Tan HIS Hoffmann KA Yiu MI Grummich KW Pharmacokinetics of coumarin and its 7-hydroxy-metabolites upon intravenous and peroral administration of coumarin in man Eur J Clin Pharmacol 1977 12 457 461 10.1007/BF00561066 598421

[B30] Tao JY Zheng GH Zhao L Wu JG Zhang XY Zhang SL Huang ZJ Xiong FL Li CM Anti-inflammatory effects of ethylacetate fraction from melilotus suaveolens Ledeb on LPS-stimulated RAW 264.7 cells Ethnopharmacol 2009 123 97 105 10.1016/j.jep.2009.02.024 19429346

